# Impact of proton-pump inhibitors on the efficacy of immune checkpoint inhibitors in non-small cell lung cancer: A systematic review and meta-analysis

**DOI:** 10.1016/j.amsu.2022.103752

**Published:** 2022-05-14

**Authors:** Sophia Dar, Nooraldin Merza, Mehek Rahim, Ahmad Qatani, Tony Varughese, Asna Mohammad, Fahad Masood, Fizza Reza, Shucenwan, Talal Almas, Aayat Ellahi, Rosario Ligresti

**Affiliations:** a Hackensack University Medical Center, Hackensack, NJ, USA; b Department of Medicine, Wayne State University, Detroit, MI, USA; c Dow University of Health Sciences, Karachi, Pakistan; d Royal College of Surgeons in Ireland, Dublin, Ireland; e Jinnah Sindh Medical University, Karachi, Pakistan

**Keywords:** Proton pump inhibitor, Immune checkpoint inhibitors, Non- small cell lung cancer, Cancer, Survival outcomes

## Abstract

**Introduction:**

Immune checkpoint inhibitors (ICI) is a rapidly evolving treatment modality for stage IV non-small cell lung cancer (NSCLC). Concomitant proton pump inhibitor (PPI) use can potentially reduce the clinical efficacy of ICIs; however, the consensus in recent literature has been conflicting. This study aims to analyze overall survival (OS) and progression-free survival (PFS) outcomes in patients with NSCLC on ICI and concomitant PPI therapy.

**Methods:**

A literature search was done in 3 databases (Pubmed/Medline, Embase, and Cochrane Central). All studies meeting the inclusion criteria assessing the impact of PPIs on the efficacy of ICI in NSCLC patients were systematically identified. A random-effects network meta-analysis evaluated OS and PFS in the two arms.

**Results:**

Four studies with 2,940 patients are included in our analysis. ICI usage alone was associated with significantly better OS [HR = 1.46, 95% CI = 1.27–1.67, P < 0.00001] and PFS [HR = 1.31, 95% CI = 1.17–1.47, P < 0.00001] when compared to concomitant PPI and ICI therapy.

**Conclusion:**

The concomitant use of PPIs during ICI therapy significantly worsens clinical outcomes with shorter OS and increased risk of disease progression in patients with NSCLC.

## Highlights

• Immune checkpoint inhibitors are a rapidly evolving treatment regime used for various cancer therapies. The role of other medications in their effectiveness remains conflicted. Proton Pump Inhibitors are one of the most commonly prescribed medications and are particularly often prescribed to cancer patients.
• Our study finds that Proton Pump Inhibitors worsen the outcomes of overall survival and increase risk of disease progression in Non-Small Cell Lung Carcinoma.
• We hope that this study will enlighten providers to use proton pump inhibitors more judiciously in patients with Non-Small Cell Lung Carcinoma.

## 1 Introduction

Combination strategies of immune checkpoint inhibitors (ICIs) that target cytotoxic T-lymphocyte antigen-4 (CTLA-4), programmed cell death protein 1 (PD-1), and its ligand (PD-L1) with standard chemotherapy agents are a rapidly evolving treatment modality for stage IV non-small cell lung cancer (NSCLC) [1,2]. However, ICI use is associated with variable responses in terms of survival and adverse outcomes even with combination approaches [3]. This fact implies that the efficacy of ICIs is essentially dependent on several modulating factors, including body composition, tumor burden, PD-L1 expression rate, and concordant medications that could exert immunomodulatory effects systemically and within the tumor microenvironment in addition to causing gut dysbiosis [4].

Proton-pump inhibitors (PPIs) have been reported to alter gut microbiota composition, thus potentially affecting the efficacy of ICIs in patients with advanced cancer [5]. They are often used in patients with cancer as long-term medication to prevent the erosion of the gastric mucosa lining caused by cancer therapy [4].

A post hoc analysis of the IMpower150 randomized clinical trial reported that PPI use was independently associated with adverse prognostic outcomes in patients with advanced NSCLC treated with atezolizumab therapy [6]. A recent meta-analysis conducted by Qin et al. also demonstrated that concomitant PPI use reduced the clinical efficacy of ICI treatment in patients with advanced cancer [7]. In contrast, previous studies have reported no significant difference in the clinical outcomes of ICIs between patients with or without concomitant PPI treatment in advanced NSCLC [8,9].

Although numerous studies have been conducted to determine the association of ICIs with concomitant PPI therapy, the reported data is conflicting. Herein, this meta-analysis aims to derive a more reliable estimate of the impact of PPIs on the efficacy of ICIs in terms of overall survival (OS) and progression-free survival (PFS) outcomes in patients with NSCLC. To the best of our knowledge, this is the first meta-analysis assessing the impact of PPI exclusively on the efficacy of ICI therapy in patients with NSCLC.

## 2 Methods

### 2.1 Literature search strategy and data sources

**Fig. 1:**
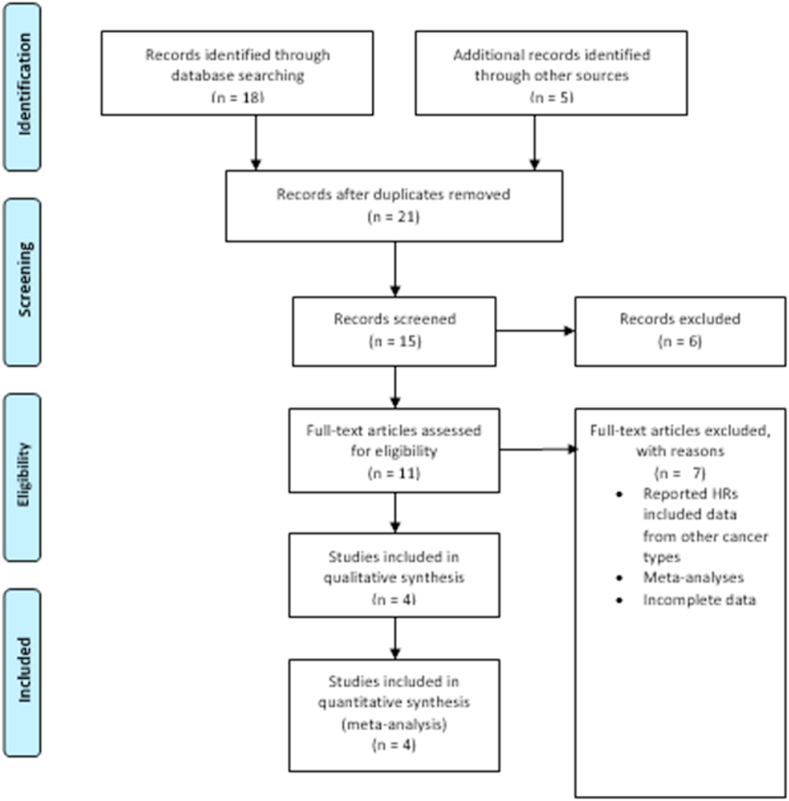
Prisma flow chart.

This systematic review and meta-analysis are reported according to the Preferred Reporting Items for Systematic Reviews and Meta-Analysis statement (PRISMA) Methods guidelines [10] (Fig. 1). Three databases (Pubmed/Medline, Embase, and Cochrane Central) were systematically searched for all relevant studies that assessed PPI's impact on the efficacy of ICI in NSCLC patients from inception through February 5th, 2022, without any time lag or language restrictions. Additional sources included bibliographies of review articles, original studies, and relevant editorials. Mesh terms and Boolean operators were used for devising an effective search strategy for each database (Supplementary Table 1). Since this study utilizes publicly available data, study registry and IRB approval were also not needed.

### 2.2 Study selection

All articles retrieved from the systematic search were exported to EndNote Reference Manager (Version X7.5; Clarivate Analytics, Philadelphia, Pennsylvania), where duplicates were removed after identification. The remaining articles were then assessed at the title and abstract level by two independent investigators (FM and FR), after which full-text articles were examined to confirm relevance. Mutual discussions with a third investigator (SD) resolved any disagreements. The following predetermined inclusion criteria were used [1]: Patient population was restricted to patients with NSCLC [2]; Studies gauging the effect of PPI on ICIs [3]; Reported data were in the form of Hazard Ratios (HR) with a 95% confidence interval or raw data through which HR could be calculated for PFS or OS. Data from post hoc analyses were also considered for inclusion. Case series, case reports, and reviews are not included, along with studies that reported data for multiple cancers.

### 2.3 Data extraction and outcomes of interest

Two investigators (FM and FZR) autonomously extracted data from the selected studies after meticulously analyzing them on pre-specified collection forms. The following information was collected from the selected articles: First author, year, population, PPI usage, study type, data type, and conclusion. HRs with 95% CIs for PFS and OS between PPI users and nonusers were also extracted with the reported follow-ups. The quality of the included studies was assessed using the Newcastle-Ottawa Quality Assessment Scale (NOS) and Cochrane Risk of bias tool for the observational studies and post hoc analyses, respectively. AMSTAR 2 criteria were used to assess the quality of the systematic review [11]. This is a high-quality review based on the AMSTAR checklist. Funding for each of the individual studies was also noted. Since there are few studies in the review, publication bias is present.

### 2.4 Statistical analyses

All statistical analyses were performed using Review Manager (version 5.4.1). The analysis was performed with the random-effects model. We present the outcomes as Hazard ratios (HRs) with 95% confidence intervals (CIs). The I^2^ statistic was used to evaluate heterogeneity across studies, and a value of I2 = 25%–50% was considered mild, 50%–75% moderate, and I^2^ >75% severe. A p-value <0.05 was considered statistically significant in all cases. The inclusion of a limited number of studies did not permit the evaluation of a publication bias.

## 3 Results

### 3.1 Search results and study characteristics

**Fig. 2:**
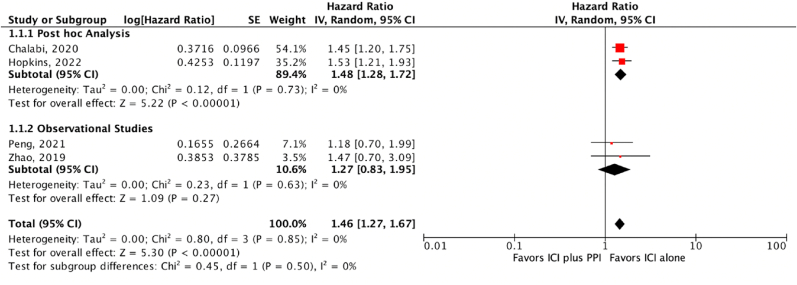
Overall survival (OS).

The initial search yielded 18 results. Eleven relevant articles were shortlisted for a full-text review after reviewing their titles and abstracts. According to our strict inclusion criteria: patient population of NSCLC, studies on PPI with ICI using hazard ratios with 95% CI or raw data to calculate HR, the number was further reduced to 4 relevant articles. The literature search by the PRISMA flow chart (Fig. 2). Unanimous agreement among authors regarding the eligibility of these studies was confirmed. A total of 2940 patients were included in our research, with a mean age of approximately 63.2 years. The mean follow-up time was calculated to be 15.4 months. Two studies were conducted globally (OAK/POPLAR study, IMpower150 study), one was restricted to China [8], and the final one presented data from the United States. (4,6,8,12) Atezolizumab (PD-L1 inhibitor) was the most common ICI used with PPIs. The ICIs in Zhao et al.'s study included nivolumab or pembrolizumab (PD-1 inhibitors), representing approximately 5% of the total patient population included in our study [8]. The baseline characteristics of the included studies are given in (Table 1).

**Table 1:** Patient characteristics.

Author	Year	Country	Sample (Y/N)	Type of PPI	Type of ICI	PPI use Window	Follow- Up Time	Study Type	Overall Survival [HR(95% CI)]	Progression Free survival [HR(95% CI)]
Zhao et. Al	2019	China	109 (40/69)	NR	Pembrolizumab, Nivolumab, Camrelizumab	Within 1 month before or after the use of ICI	28 months	RC	1.47(0.70–3.06), P = 0.309	1.10(0.65–1.85), P = 0.725
Chalabi et al.	2020	Worldwide	757 (235/522)	Omeprazole, Pantoprazole, L ansoprazole, R abeprazole, E someprazole, D exlansopra zole	Atezolizumab	Within 30 days before or after the start of ICI	19.2 months	RCT	1.45 (1.20–1.75), P = 0.0001	1.30(1.10–1.53), P = 0.001
Peng et al.	2021	United States	117 (46/71)	Omeprazole, P antoprazole, E someprazole, D exlansoprazole, or multiple PPIs	Pembrolizumab, Nivolumab	Within 30 days before or after the start of ICI	12 months	RCT	1.18 (0.70, 2.01)	1.33 (0.86, 2.04)
Hopkins et. Al	2021	Worldwide	1202 (441/761)	Omeprazole, P antoprazole, E someprazole, L ansoprazole, D exlansoprazole, R abeprazole	Atezolizumab, B evacizumab	Within 30 days before or after the start of ICI	40 months	RCT	1.53 (1.21–1.95), P < 0.001	1.34 (1.12–1.61), P = 0.002

Y/N, PPI use/no use.

RC = Retrospective Cohort.

RCT = Randomized Control Trial.

### 3.2 Quality assessment

According to the NOS quality assessment criteria, both included retrospective studies that indicated low bias, with scores of 8 and 9 for Peng et al. and Zhao et al., respectively. As per the Cochrane risk of bias assessment tool, the two RCTs whose post analyses were included exhibited high bias according to the Cochrane risk bias assessment tool. The heterogeneity of the studies for OS and PFS were both 0%. The detailed information is present in Supplementary Table 1, Supplementary Table 2, Supplementary Fig. 1a, and Supplementary Fig. 1b.

### 3.3 OS

Our meta-analysis concluded that concomitant ICI and PPI is associated with a worse clinical outcome with a shorter OS compared to patients on ICI without PPI [HR = 1.46, 95% CI = 1.27–1.67, P < 0.05] (Fig. 2).

### 3.4 PFS

**Fig. 3:**
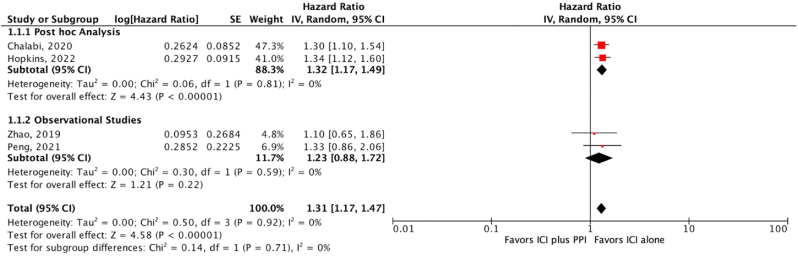
Progression free survival (PFS).

Our results found that concurrent PPI and ICI increase the risk for the progression of the disease. When compared to patients on ICI without PPI. [HR = 1.31, 95% CI = 1.17–1.47, P < 0.05] (Fig. 3).

## 4 Discussion

This study is the most updated meta-analysis, including the latest research on the effect of PPI on ICI therapy in NSCLC patients. We found that PPI are associated with shorter OS and increased risk of disease progression. Our analysis is consistent with the outcome trends in the two included post hoc analyses of the OAK/POPLAR and IMpower150 trials. In the pooled post hoc analyses of OAK and POPLAR trials, OS and PFS were significantly shorter for patients who used concomitant PPI in the atezolizumab group than those who had not [12]. The post hoc analysis of IMpower150 demonstrated that PPI use was independently associated with worse clinical endpoints in the pooled arms of atezolizumab [6]. Interestingly, the study described that PPI is associated with reduced OS. A greater risk of progression was seen in the immunotherapy arm but not the chemotherapy control arms, which underscores a potential modulatory effect of PPIs on the mechanism of action or clinical efficacy of ICI.

Conversely, data from Peng et al. showed that the current use of PPI in advanced cancer patients treated with nivolumab or pembrolizumab was not significantly associated with an impact on OS and PFS [4]. Likewise, results from Zhao et al. suggest a non-significant difference in ICI efficacy with concurrent PPI use in patients with NSCLC [8]. One explanation for this discrepancy is that the sample size for both mentioned studies is relatively small and thus not powered to detect the impact of PPI on ICI. Furthermore, by the nature of the study design, observational data cannot differentiate between causation and association in complex outcomes (OS and PFS) and thus must be interpreted with caution.

The previous meta-analysis by Qin et al. showed that concomitant PPI use is associated with lower clinical efficacy of ICIs in patients with advanced cancer in terms of survival outcomes which is consistent with the findings of our meta-analysis [7]. However, a meta-analysis by Li et al. reported an intriguing discovery demonstrating that PPIs had variable effects in different cancer types, with concurrent PPI therapy showing a detrimental effect on NSCLC patients but a positive effect on melanoma patients [13]. The variation in concurrent PPI response is attributed mainly to the alteration in the abundance of taxa which are critical determinants of the clinical efficacy of ICI.

The potential for PPIs to attenuate the effects of ICI is multifactorial. One potential mechanism is their direct suppression of different steps in the immunological cascade, including reducing the expression of adhesion molecules by immune cells and manipulating the secretions of proinflammatory cytokines*.* [14] Previous studies have demonstrated that patients with a more diverse gut microbiome positively respond to ICI treatment [15,16]. PPI use is associated with a reduction of gut microbiota diversity and diminishes the relative abundances of Ruminococcaceae/Faecalibacterium spp, leading to a relative decrease in the antitumor immune activity of patients on PPI therapy [17,18]. The proposed mechanism led to the hypothesis that concurrent PPI use affects the production of specific immunological cells and induces alteration in gut microbiota, decreasing ICI's clinical efficacy. The findings of this meta-analysis align with this notion.

PPI are one of the most commonly prescribed medications globally due to its favorable safety profile and robust efficacy [19,20]. PPI regimen is also frequently used along with cancer therapy [21]. Various factors determine the necessity for PPI in patients receiving ICI treatment. Patients taking non-steroidal anti-inflammatory drugs (NSAIDs) as analgesics for cancer pain were routinely given PPI as a preventative measure. Cancer patients are also known to have a higher risk of peptic ulcer disease (PUD) or its complications, and those with a history of PUD or advanced age were frequently given PPI for prophylactic use [22].

In the light of current evidence, the relationship between the impact of concurrent medications on the microbiome and immunotherapy response appears to be strengthening. Based on the AMSTAR checklist, this is a high-quality review. Funding for each of the individual studies was also noted. Since there are few studies in this review, publication bias is present [23].

There are several limitations to this meta-analysis that should be noted. First, selection and reporting bias is inherently present in the retrospective studies due to lack of randomization. In addition, the post hoc analyses included in our study comprised of un-blinded RCTs, leading to overestimation of treatment effects and performance and detection bias. Similarly, due to the relatively smaller sample size of the observational studies, the possibility of extrapolating results cannot be ignored. Specific studies were also eliminated because they lacked critical information to calculate HR for OS or PFS. However, this particular selection may have an unpredictably negative impact on our outcomes. Importantly, due to a lack of precise information, the heterogeneity resulting from timing, different types, dosages, and compliance to PPI administered has not been evaluated. Finally, the current data surrounding this topic presents the potential for confounders. For instance, whether PPIs are prescribed to an inherently sicker population and the frequent co-administration of corticosteroids with PPIs may skew their actual effect. Thus, these ideas would need to be further evaluated in large randomized controlled trials.

## 5 Conclusion

In this study, we evaluated the effect of PPI on the efficacy of ICI in patients with NSCLC through a meta-analysis and systemic review of the current literature. PPI use with ICI resulted in shorter OS and increased risk of disease progression. Our study findings make it crucial for clinicians to carefully and cautiously assess the indication for PPI in patients who are either candidate for ICI therapy or are currently on ICI therapy for NSCLC.

## Ethical approval

This study was conducted on publicly available data previously published and therefore did not require an IRB.

## Sources of funding for your research

There is no finding for this research.

## Author contribution

Sophia Dar-study design and concept, analysis, writing and finalizing manuscript Nooraldin Merza-analysis, writing manuscript Mehek Rahim-analysis, writing manuscript Ahmad Qatani-data extraction, writing and editing manuscript Tony Varughese-data extraction, writing and editing manuscript Asna Mohammed-data extraction, writing and editing manscript Fahad Masood-data search and writing manuscript Fizza Reza-data search and writing manuscript Schuchen Wan-data search and writing manuscript Talal Almas-editing manuscript Aayat Ellahi-editing manuscript Rosario Ligresti-editing and finalizing manuscript and contributing to study design and concept.

## Conflicts of interest

No conflicts of interest.

## Consent

N/A.

## Registration of research studies

1. Name of the registry: N/A
2. Unique Identifying number or registration ID:
3. Hyperlink to your specific registration (must be publicly accessible and will be checked):

## Guarantor

Sophia Dar and Talal Almas.
