# Supplementary material for: Impact of proton-pump inhibitors on the efficacy of immune checkpoint inhibitors in non-small cell lung cancer: A systematic review and meta-analysis
Source: Ann Med Surg (Lond). 2022 May 14;78:103752. doi: 10.1016/j.amsu.2022.103752 (PMC9119820; doi:10.1016/j.amsu.2022.103752)

**Supplementary material**

| **Supplemental Table 1**. Search strategy used in each database searched | |  |
| --- | --- | --- |
| **Database (Articles Retrieved)** | **Search Strategy** |  |
| PUBMED (18 results) | ("carcinoma, non small cell lung"[MeSH Terms] OR ("carcinoma"[All Fields] AND "non small cell"[All Fields] AND "lung"[All Fields]) OR "non-small-cell lung carcinoma"[All Fields] OR ("non"[All Fields] AND "small"[All Fields] AND "cell"[All Fields] AND "lung"[All Fields] AND "carcinoma"[All Fields]) OR "non small cell lung carcinoma"[All Fields] OR ("carcinoma, non small cell lung"[MeSH Terms] OR ("carcinoma"[All Fields] AND "non small cell"[All Fields] AND "lung"[All Fields]) OR "non-small-cell lung carcinoma"[All Fields] OR ("non"[All Fields] AND "small"[All Fields] AND "cell"[All Fields] AND "lung"[All Fields] AND "cancer"[All Fields]) OR "non small cell lung cancer"[All Fields]) OR ("carcinoma, non small cell lung"[MeSH Terms] OR ("carcinoma"[All Fields] AND "non small cell"[All Fields] AND "lung"[All Fields]) OR "non-small-cell lung carcinoma"[All Fields] OR "nsclc"[All Fields] OR "nsclc s"[All Fields] OR "nsclcs"[All Fields])) AND ("immune checkpoint inhibitors"[Pharmacological Action] OR "immune checkpoint inhibitors"[MeSH Terms] OR ("immune"[All Fields] AND "checkpoint"[All Fields] AND "inhibitors"[All Fields]) OR "immune checkpoint inhibitors"[All Fields] OR "ICI"[All Fields] OR ("immune checkpoint inhibitors"[Pharmacological Action] OR "immune checkpoint inhibitors"[MeSH Terms] OR ("immune"[All Fields] AND "checkpoint"[All Fields] AND "inhibitors"[All Fields]) OR "immune checkpoint inhibitors"[All Fields] OR "pd 1 inhibitors"[All Fields]) OR ("immune checkpoint inhibitors"[Pharmacological Action] OR "immune checkpoint inhibitors"[MeSH Terms] OR ("immune"[All Fields] AND "checkpoint"[All Fields] AND "inhibitors"[All Fields]) OR "immune checkpoint inhibitors"[All Fields] OR ("pd"[All Fields] AND "l1"[All Fields] AND "inhibitors"[All Fields]) OR "pd l1 inhibitors"[All Fields]) OR ("immune checkpoint inhibitors"[Pharmacological Action] OR "immune checkpoint inhibitors"[MeSH Terms] OR ("immune"[All Fields] AND "checkpoint"[All Fields] AND "inhibitors"[All Fields]) OR "immune checkpoint inhibitors"[All Fields] OR "ctla 4 inhibitors"[All Fields]) OR ("pembrolizumab"[Supplementary Concept] OR "pembrolizumab"[All Fields]) OR ("nivolumab"[MeSH Terms] OR "nivolumab"[All Fields] OR "nivolumab s"[All Fields]) OR ("cemiplimab"[Supplementary Concept] OR "cemiplimab"[All Fields]) OR ("ipilimumab"[MeSH Terms] OR "ipilimumab"[All Fields]) OR ("atezolizumab"[Supplementary Concept] OR "atezolizumab"[All Fields]) OR ("durvalumab"[Supplementary Concept] OR "durvalumab"[All Fields]) OR ("avelumab"[Supplementary Concept] OR "avelumab"[All Fields])) AND ("proton pump inhibitors"[Pharmacological Action] OR "proton pump inhibitors"[MeSH Terms] OR ("proton"[All Fields] AND "pump"[All Fields] AND "inhibitors"[All Fields]) OR "proton pump inhibitors"[All Fields] OR "PPIs"[All Fields] OR ("omeprazole"[MeSH Terms] OR "omeprazole"[All Fields] OR "esomeprazole"[MeSH Terms] OR "esomeprazole"[All Fields] OR "omeprazol"[All Fields]) OR ("pantoprazole"[MeSH Terms] OR "pantoprazole"[All Fields] OR "pantoprazol"[All Fields]) OR ("esomeprazole"[MeSH Terms] OR "esomeprazole"[All Fields] OR "esomeprazol"[All Fields]) OR ("lansoprazole"[MeSH Terms] OR "lansoprazole"[All Fields] OR "lansoprazol"[All Fields]) OR ("dexlansoprazole"[MeSH Terms] OR "dexlansoprazole"[All Fields]) OR ("rabeprazol"[All Fields] OR "rabeprazole"[MeSH Terms] OR "rabeprazole"[All Fields])) |  |
|  |  |  |
| Cochrane Central (5 results) | (Non-small cell lung carcinoma OR Non-small cell lung cancer OR NSCLC) AND (Immune checkpoint inhibitors OR ICI OR PD-1 inhibitors OR PD-L1 inhibitors OR CTLA-4 inhibitors OR Pembrolizumab OR Nivolumab OR Cemiplimab OR Ipilimumab OR Atezolizumab OR Durvalumab OR Avelumab) AND (Proton pump inhibitors OR PPIs OR Omeprazole OR Pantoprazole OR Esomeprazole OR Lansoprazole OR Dexlansoprazole OR rabeprazole) |  |
|  |  |  |
|  |  |  |

| **Supplemental Table 2**. Quality Assessment of included observational studies using the New Castle-Ottawa Scale | | | | | | | | | |
| --- | --- | --- | --- | --- | --- | --- | --- | --- | --- |
|  | | | | | | | | | |
|  | **Selection** | | | | **Comparability** | **Outcome** | | |  |
| **Study** | **Representativeness of the exposed cohort** | **Selection of the non-exposed cohort** | **Ascertainment of exposure** | **Demonstration that the current outcome of interest was not present at start of the study** | **Comparability of cohorts on the basis of the design or analysis** | **Assessment of outcome** | **Was follow-up long enough for outcomes to occur** | **Adequacy of follow-up of cohorts** | **Quality Score** |
| Zhao, 2019 | * | * | * | * | ** | * | * | * | 9 |
| Peng, 2021 | * | * | * | * | * | * | * | * | 8 |

**Supplemental Figure 1a**. Quality Assessment of included Randomized Controlled Trials using Cochrane Risk of Bias Tool

**Risk of bias graph**


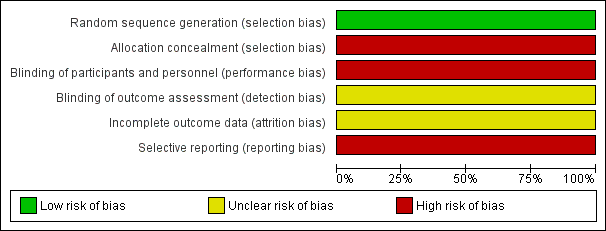


**Supplemental Figure 1b.** Quality Assessment of included Randomized Controlled Trials using Cochrane Risk of Bias Tool

**Risk of bias Summary**


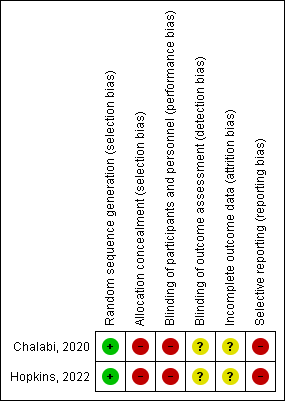


**Supplemental Figure 2**. PRISMA Flow Diagram


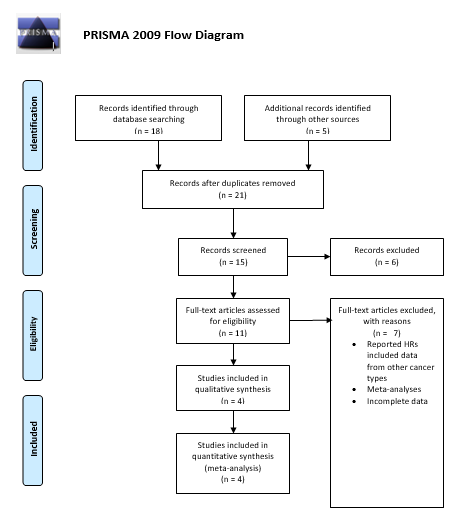

Supplement: Multimedia component 3 [file mmc3.docx]
